# Concealed complete response in melanoma patients under therapy with immune checkpoint inhibitors: two case reports

**DOI:** 10.1186/s40425-017-0309-3

**Published:** 2018-01-15

**Authors:** Stefan Schliep, Abbas Agaimy, Alexander Cavallaro, Franklin Kiesewetter, Gerold Schuler, Lucie Heinzerling

**Affiliations:** 1 Department of Dermatology, University Hospital Erlangen, Ulmenweg 18, 91054 Erlangen, Germany; 2 Institute of Pathology, University Hospital Erlangen, Krankenhausstr. 8-10, 91054 Erlangen, Germany; 3 Institute of Radiology, University Hospital Erlangen, Ulmenweg 18, 91054 Erlangen, Germany

**Keywords:** Melanoma, Complete response, Immune checkpoint, Checkpoint inhibitor, Pseudoprogression

## Abstract

**Background:**

The assessment of tumor size by RECIST using CT scans and MRIs is considered to be standard of care for staging cancer patients. Despite radiologic evidence of widespread disease, we document for the first time that patients were completely free of viable tumor.

**Case presentation:**

Two patients with metastatic melanoma were treated with immune checkpoint inhibitors (ipilimumab/ nivolumab) and progressive metastases were detected on CT-scans performed shortly before histologic examinations. In both patients histologic assessment revealed a complete response with necrotic and scarred lesions free of tumor. One of the patients had started immunotherapy 20 months before with an initial partial response.

**Conclusions:**

This phenomenon of a concealed complete response can lead to overtreatment or unnecessary change in treatment. Thus, it is essential to raise awareness for it. Correct identification of responders to immune checkpoint inhibitor therapy is crucial to spare patients immune-mediated side effects and unnecessary as well as expensive treatment. Regression of metastases without decline in size, in these cases manifesting as complete responses, are probably more common than expected and identified to date. Until such responses can be readily identified by new imaging techniques, we recommend liberal biopsies for histologic assessment of progressive metastases in patients during and/or after immune checkpoint inhibitor therapy.

## Background

Checkpoint inhibitors are effective in the treatment of metastatic melanoma, with approval of the first antibodies in the U.S. in 2011 [1]. Ipilimumab, the anti-CTLA4 antibody, demonstrated an increased overall survival [2]. Nivolumab and pembrolizumab, both antibodies directed against PD1, displayed even higher response rates than ipilimumab and also an improved overall survival [3, 4], but the highest response rate of melanoma patients so far was seen in a combination of ipilimumab and nivolumab [5]. A major drawback of the therapy with immune checkpoint inhibitors is a variety of side effects, most of which are immune-mediated [6]. Besides these improved treatment outcomes and new side effect profiles novel response patterns have already been observed in the phase II program. These led to the development of specific radiologic “immune-related response criteria” [7], that complement the established RECIST 1.1 criteria [8]. It became evident that partial or complete responses to therapy can develop after an initial increase of tumor burden in imaging studies – a phenomenon called pseudoprogression. It is crucial to note that an increase of tumor burden > 25% in a control examination after 4 weeks is regarded as definite progression. This also applies to the two patients presented here, who had progressive disease assessed according to irRC as well as RECIST 1.1. Surprisingly, the histopathologic examination of progressive metastases shortly after imaging proved them to be totally free of viable tumor cells.

## Case presentation #1

A 72-year old patient had a history of a nodular melanoma (T4b) on the left forearm, followed by an excision with a safety margin of 2 cm and a sentinel lymph node biopsy (0/1). While on treatment with adjuvant low-dose interferon-alpha (3 × 3 Mio IE/week) a lymph node metastasis in the left axilla was diagnosed, followed by axillary lymph node dissection. A year later distant lymph node metastases were recognized and confirmed via exstirpation with histologic examination.

Additionally, the patient suffered from coronary artery disease with a myocardial infarction and bypass surgery in 2007 but normal ejection fraction assessed in 2011.

**Fig. 1:**
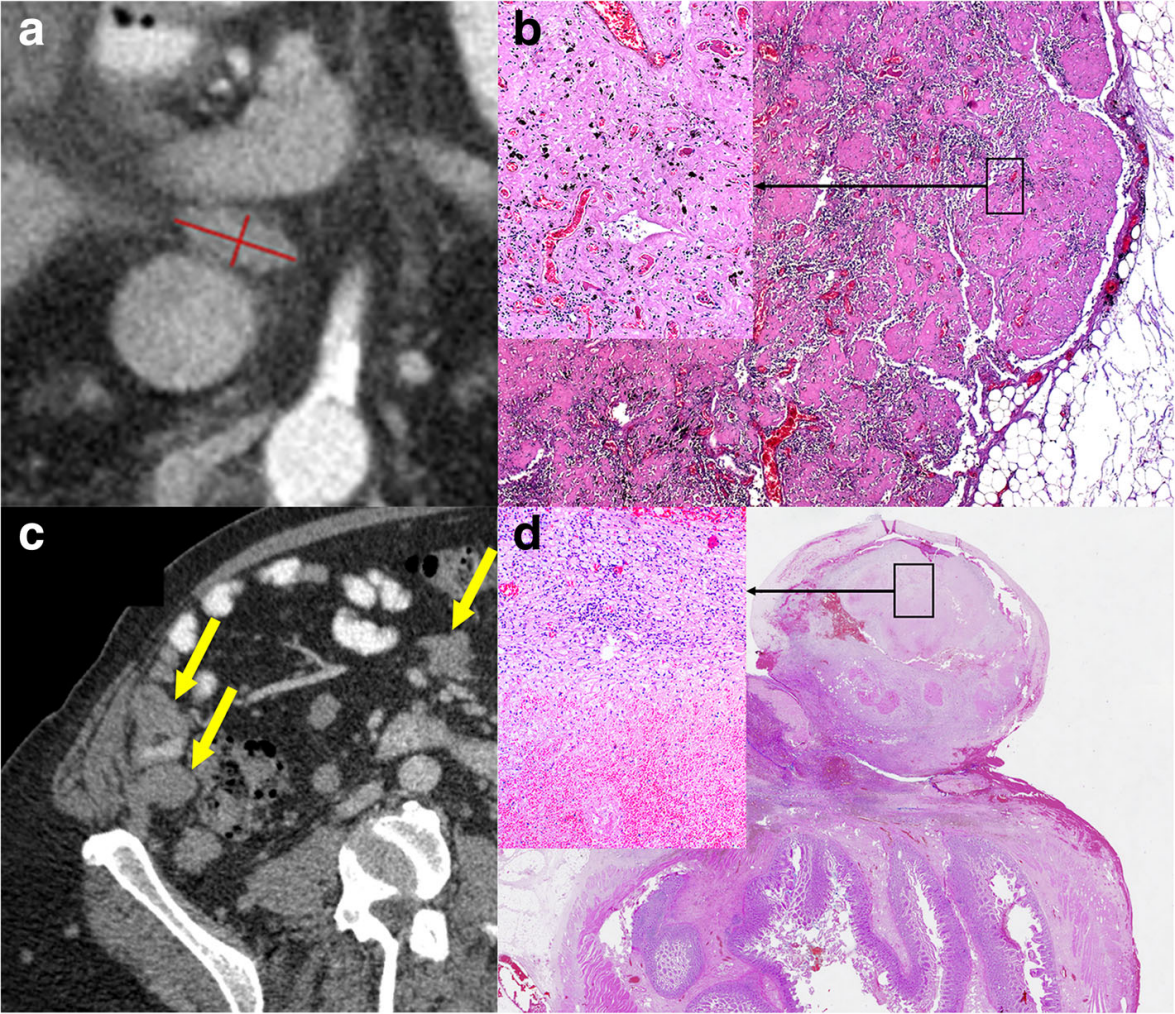
CT scans indicating metastatic disease and corresponding tumor-free histologic evaluation after therapy with checkpoint inhibitors. In Patient 1, a 72-year-old man with metastatic melanoma a partial response was seen initially after checkpoint inhibitor therapy. Then, lymph node metastases again progressed (progress of 28% as assessed by Recist 1.1; Panel **a**). On autopsy after death due to cardiac failure these nodes showed no evidence for viable tumor tissue (Panel **b**). In patient 2 multiple tumor nodes were seen to increase in size in the mesenterial fat and in vicinity of the bowel (Panel **c**, arrows). Histologic analysis due to a pathologic fracture and a perforation showed no viable tumor tissue as shown in the representative lesion of the ileum (Panel **d**)

Furthermore, he had type 2 diabetes mellitus, hypertension, arterial obstructive disease of the legs and colon polyposis. The patient was enrolled in a checkpoint inhibitor trial (CA 209067) in 2013 and initial imaging showed cervical, supraclavicular, mediastinal, hilar and abdominal lymph node metastases. He received 4 infusions of ipilimumab (3 mg/kg body weight) combined with nivolumab (1 mg/kg body weight) followed by another 5 infusions of nivolumab at the dose of 3 mg/kg body weight every two weeks. Staging revealed a partial response with a nadir of the RECIST sum of 1.5 cm compared to 5.5 cm at baseline. Due to cardiomyositis with a reduced ejection fraction (EF) of 15% treatment was interrupted. Since myocardial biopsy was consistent with immune-mediated changes, corticosteroids were initiated and improved EF within days. The patient remained stable for 1 year after cessation of treatment. Then, however, progressive disease was diagnosed with increasing cervical, mediastinal, hilar and abdominal nodes in radiologic imaging (Fig. 1 a) and the patient received pembrolizumab. Subsequently, he developed a severe cardiomyopathy, and died 2 months later due to cardiac decompensation. Autopsy was performed, and surprisingly the pathologic examination of all grossly suspected metastatic lesions showed extensive necrosis with hyalinization, calcification and mixed inflammatory infiltration with variable anthracotic changes without any viable tumor cells were detected (ypT0 ypM0 L0 V0 Pn0; Fig. 1 b). Furthermore, clinical diagnosis of cardiomyopathy was confirmed in the autopsy without evidence for autoimmune myocarditis.

## Case presentation #2

A 64-year old male patient presented with BRAF wildtype metastatic melanoma 7 months after resection of a T4b nodular melanoma of the scalp without involvement of the sentinel lymph node (0/1). Staging revealed widespread disease with recurrence of metastases at the scalp and liver, lung, and lymph node metastases (neck, both axillae, mediastinum, lung hilus). After palliative surgery of the rapidly growing metastases at the scalp imaging showed progressive disease with increasing lung, liver, renal and bone metastases as well as extensive lymph node and soft tissue metastases (Fig. 1 c). The patient was treated with nivolumab and ipilimumab. After the first treatment he developed autoimmune colitis with CTCAE grade 1 diarrhea. After four infusions, he was admitted for osteosynthetic management of a pathologic femur fracture due to bone metastases. While still hospitalized he developed massive abdominal pain. A perforation was diagnosed with free air on abdominal X-ray and the patient underwent surgery. Surprisingly, histopathologic analyses of the resected ileum with the tumor masses and suspected mesenteric lymph node metastases as well as of the fractured bone showed no evidence of vital tumor. The resected ileum tumor exhibited an intramural necrotic, scarred lesion with macrophages and siderophages (Fig. 1 d), without detectable melanoma cells or lymphocytic infiltrates. Therefore, we postulate that necrotic metastases led to the perforation, rather than an immune-mediated colitis. Treatment with nivolumab was continued. However, the patient died from pneumonia and subsequent sepsis despite immediate treatment. Pneumonitis was excluded by CT scan. Unfortunately, no autopsy was done.

## Discussion and conclusions

Currently, tumor response is evaluated based on tumor size on CT and MR scans [7, 8]. FDG-PET-CT provides greater sensitivity than CT and also specificity since it can detect early changes in metabolism which might precede morphological changes [9]. This has been shown, e.g. in gastrointestinal stromal tumors treated with imatinib where response on PET was associated with PFS and preceded tumor shrinkage by several weeks [10]. Successful checkpoint inhibitor therapy can induce immune cell infiltration with a subsequent increase in tumor volume [11], i.e. pseudoprogression, which can be followed by a partial or complete response. This observation led to the development of the immune-related response criteria (irRC, 7) which define progression as an increase of tumor burden of at least 25% at 2 different time points with an interval of at least 4 weeks [7]. Both patients had progressive disease in the CT scans shortly before pathologic examinations revealed scarred, tumor-free metastases even when applying immune-related response criteria.

The concealed response was seen just 6 weeks after initiation of checkpoint inhibitor treatment in patient #2, whereas patient #1 had progressive nodes more than a year after start of and response to therapy. This demonstrates that concealed regression of metastases can be induced very early under immune checkpoint inhibitor therapy and (pseudo-)progression in imaging studies can occur as late as 15 months after start of treatment despite histologically confirmed response. In case #2, vital tumor manifestations at other sites could not be excluded since only abdominal and bone tumors were pathologically assessed. Concealed responses in patients that show stable or progressive tumors on radiologic staging may be much more common than expected and identified under immunotherapy with checkpoint inhibitors. Importantly, there have been two cases of radiologic pseudoprogression of brain metastases in patients treated with PD1-antibodies [12, 13]. Pathologic similarities with necrotic tissue and no vital tumor were reported in a lung carcinoma patient who underwent radiotherapy more than 2 years before anti-PD1 treatment [12]. In contrast, vital tumor with treatment-related CD45^+^ and CD68^+^ infiltrates was seen in a BRAF^V600E^-mutant melanoma patient with multiple small brain metastases after a single application of pembrolizumab [13].

In order to spare patients potential toxicity of unnecessary treatment or (even worse) change of an effective therapy in responding patients innovative methods for the detection of viable tumor tissue are desperately needed. FDG-PET/CT in melanoma could be valuable at least in sclerosed lesions but not in lesions heavily infiltrated by immune cells which due to their metabolism will be FDG-PET positive [14].

A prospective study with histologic assessment of ‘metastases’ under treatment with checkpoint inhibitors should be considered to confirm our findings. In tumor patients treated with immune checkpoint inhibitors progressive tumor manifestations may represent a concealed response without viable tumor. If suspected only histology can solve the diagnostic challenge.
